# Acceptability of Internet-based interventions for problem gambling: a qualitative study of focus groups with clients and clinicians

**DOI:** 10.1186/s12911-019-1011-9

**Published:** 2019-12-30

**Authors:** Sherald Sanchez, Farah Jindani, Jing Shi, Mark van der Maas, Sylvia Hagopian, Robert Murray, Nigel Turner

**Affiliations:** 1 Institute for Mental Health Policy Research, Centre for Addiction and Mental Health, 33 Russell Street, Toronto, ON M5S 2S1 Canada; 2 Dalla Lana School of Public Health, University of Toronto, 155 College Street, Toronto, Ontario, M5T 1P8 Canada; 3 Rehabilitation Sciences, University of Toronto, 500 University Avenue, Toronto, Ontario, M5G 1V7 Canada

**Keywords:** Gambling, Treatment, Intervention, Internet, Online, eMental health, Focus groups

## Abstract

**Background:**

Although Internet-based interventions (IBIs) have been around for two decades, uptake has been slow. Increasing the acceptability of IBIs among end users may increase uptake. In this study, we explored the factors that shape acceptability of IBIs for problem gambling from the perspective of clients and clinicians. Findings from this qualitative study of focus groups informed the design and implementation of an IBI for problem gambling.

**Methods:**

Using a semi-structured interview guide, we conducted three focus groups with clients experiencing gambling problems (total *n* = 13) and two with clinicians providing problem gambling treatment (total *n* = 21). Focus groups were audio recorded, transcribed verbatim, and analyzed using a two-part inductive-deductive approach to thematic analysis.

**Results:**

Although both user groups reported similar experiences, each group also had unique concerns. Clinician perspectives were more homogeneous reflective of healthcare professionals sharing the same practice and values. Clinicians were more concerned about issues relating to the dissemination of IBIs into clinical settings, including the development of policies and protocols and the implications of IBIs on the therapeutic relationship. In comparison, client narratives were more heterogeneous descriptive of diverse experiences and individual preferences, such as the availability of services on a 24-h basis. There was consensus among clients and clinicians on common factors influencing acceptability: access, usability, high quality technology, privacy and security, and the value of professional guidance.

**Conclusions:**

Acceptability is an important factor in the overall effectiveness of IBIs. Gaining an understanding of how end users perceive IBIs and why they choose to use IBIs can be instrumental in the successful and meaningful design, implementation, and evaluation of IBIs.

## Background

To date, research in the utility and impact of Internet-based interventions (IBIs) in the treatment of mental health issues, particularly for depression and anxiety [1, 2], have shown positive effects. There is interest in seeing this success replicated in the field of addictions [3–5], including problem gambling [6–8].

Problem gamblers report several barriers to treatment including feelings of shame and embarrassment, stigma, denial, and a desire to solve the problem on their own [9, 10]. Treatment cost, availability, and effectiveness are also commonly cited barriers [9–11], as well as geographical and time constraints [10]. Treatment delivery through IBIs can mitigate the negative impacts of these barriers. A recent scoping review of IBIs for problem gambling [6] concluded that they are a promising direction in preventing and treating problem gambling, as well as in reducing barriers to accessing professional help. Though highly valuable, these findings offer little explanation as to how users, typically healthcare providers and their clients, perceive and engage with IBIs.

Acceptability is increasingly acknowledged to be an important domain to consider in the design, implementation, and evaluation of healthcare interventions, including Internet-based interventions (IBIs). Most healthcare interventions are complex in nature. Developers are constantly faced with the challenge of designing effective interventions that maximize the resources available to deliver optimal clinical outcomes [12]. With the release of the first World Health Organization guideline on digital health interventions (DHIs) earlier this year, the role of digital health in health system strengthening and universal health coverage was highlighted [13]. IBIs and DHIs are often used interchangeably in the literature, however, DHI is a broader term encompassing both IBIs and non-internet computer-based interventions. Despite the collective recognition of its value, “digital health has also been characterized by implementations rolled out in the absence of a careful examination of the evidence base on benefits and harms [13].” In its list of evidence-based recommendations, the guideline identified acceptability as a facilitator to successful uptake and implementation. This finding is supported by recent research evaluating the design, implementation, and impact of DHIs [14–17]. Despite this, research assessing the acceptability of IBIs is very limited.

Heeding the recommendations advanced in the WHO guideline [13], we conducted a series of focus groups with clinicians and clients of a problem gambling treatment clinic in Toronto, Canada. We used the definition of acceptability provided by Sekhon and colleagues [12], which described it as “a multi-faceted construct that reflects the extent to which people delivering or receiving a healthcare intervention consider it to be appropriate, based on anticipated or experienced cognitive and emotional responses to the intervention.” The authors reasoned that assessing the acceptability prior to participation in the intervention can highlight aspects that can be modified to increase acceptability, and thus uptake [12].

Focus group objectives were two-fold: 1) to gain a better understanding of the barriers experienced by gamblers in accessing treatment, and 2) to understand how clients and clinicians perceive IBIs for problem gambling. The focus groups were a part of a larger multi-stage mixed methods study exploring the feasibility of an online treatment service for problem gambling. Findings informed the design, implementation, and evaluation of a pilot IBI for problem gambling that concluded in June 2019.

## Methods

We explored the concept of acceptability of IBIs for problem gambling through semi-structured focus groups with clients and clinicians. In health services research, focus group methods are useful in examining patient experiences and exploring the attitudes and needs of healthcare professionals [18]. Focus groups enable participants to become an active part of the design process. As end users, gaining the perspectives of clients and clinicians is a crucial part to successful intervention design in healthcare settings [12, 19, 20]. Data saturation was reached after the second focus group with each end user group.

### Design and sampling

Between June 2017 and January 2018, three focus groups with clients (total *n* = 13) and two focus groups with clinicians (total *n* = 21) were conducted in Toronto, Canada. The study was approved by the Centre for Addiction and Mental Health (CAMH) research ethics board. Using purposive and snowball sampling techniques, clinicians were recruited by the researchers, while clients were recruited by the researchers or by clinicians at CAMH and partner agencies in the Greater Toronto Area. To ensure rigour and data saturation, we aimed to have four to eight participants in each focus group [21].

### Participants

Among the clinicians who participated in the focus groups, five were males, while the rest were females. This group was comprised of seven social workers, nine social service workers, three community health and education specialists, a psychotherapist, and an occupational therapist. Clinicians in attendance were from eight organizations across Ontario, Canada working in the problem gambling treatment sector at the time. The clients in the focus groups were adults living in the Greater Toronto Area in Ontario, Canada who self-reported having gambling problems. Among the clients attending the first two focus groups, only one was female. To gain female perspectives, a third female-only focus group was conducted in which three female clients participated.

### Focus groups

Focus groups were conducted using a semi-structured discussion guide that was developed through an iterative process, whereby data from transcripts and researchers’ notes from the first focus group were examined and used to guide the discussion in subsequent focus groups. Each focus group started with an introduction of the study. All participants provided informed consent and gave permission for audio recording. Focus groups lasted from 38 to 77 min. A third client focus group was added exclusively for female participants to address the lack of female representation in the prior focus group discussions. This last client focus group was also important in addressing issues related to gender differences as males tend to dominate during focus group discussions [22].

### Data analysis

Focus groups were audio recorded, transcribed verbatim, and analyzed in two parts:

### Part 1.1 inductive thematic analysis

This process started with reading all data repeatedly to achieve immersion and obtain a sense of the whole. Then, codes were derived by highlighting key concepts and identifying patterns. Next, codes were sorted into themes based on how different codes are related and linked. These emergent themes were reviewed extensively before they are grouped and organized into meaningful categories, which were then defined and named. This process resulted into Tables 2, 3, and 5.

**Table 1:** Items included in the semi-structured discussion guides

Focus groups with clients	Focus groups with clinicians
Introduction of the study – moderators identify themselves and explain the goals of the focus groups and the nature of the study	Introduction of the study – moderators identify themselves and explain the goals of the focus groups and the nature of the study, clinicians introduce themselves
Experience with supports and services – What are supports and services you have accessed for your gambling problems?	Experience in providing treatment support remotely – Describe your experience in providing treatment support remotely.
Barriers to accessing existing services – What gets in the way of accessing services?	Lessons from existing services – What do we know from existing services? How can we translate in-person services into online services?
Acceptability of Internet-based interventions for problem gambling – Would it be helpful if existing services were provided online? What services would be useful if provided online?	Acceptability of Internet-based interventions for problem gambling – What do you see as possible advantages and disadvantages of online services versus in-person services?
Desired features – What would you like to see in an online treatment service?	Perceived challenges and proposed solutions – What challenges and issues do you expect to encounter in providing treatment support over the Internet? What are ways we can address these issues?
Themes identified from pilot focus group – How important is it to receive support in a female-only environment? How important are clinicians with lived experience? How willing would you be to download or install a program on your computer or mobile phone device?	Implementation and future direction – What kind of supervision and professional development would help you feel comfortable working with clients online?

### Part 1.2 deductive thematic analysis

We identified and grouped similar data into a priori themes derived from the theoretical framework of acceptability (TFA) developed by Sekhon and colleagues [12]. The TFA has the benefit of being applicable in assessing acceptability of healthcare interventions both prospectively (i.e., anticipated or perceived) and retrospectively (i.e., experienced) from the perspective of those delivering and receiving the intervention. Table 4 has a complete list of all themes adopted from the TFA.

Thematic analysis is useful in gaining understanding people’s experiences [23]. Representative quotes for each theme and sub-theme were extracted and reported. Data was coded from transcripts with the aid of qualitative data management software NVivo 11.4.7 (QSR International). Two of the authors (SS, JS) independently developed a coding scheme for two transcripts by identifying, classifying, and labelling the primary patterns in the content. Interrater reliability between the two coders ranged from 90.49 to 100% – measured using Kappa coefficient on NVivo 11. The first author coded all remaining transcripts.

## Results

In this study, we aimed to understand the factors that shape the acceptability of IBIs for problem gambling among end user groups (‘If this service existed, would you use it? Why or why not?’); and to identify factors that can increase the acceptability of IBIs for problem gambling (‘What would you like to see in this type of service?’). From the focus groups, we learned that it is important to recognize the motivations behind the choice to use IBIs in order to understand the factors that influence and increase the acceptability of IBIs for clients and clinicians. Our findings are thus reported in this order.

### Part 1 motivations for using IBIs

Clients and clinicians identified motivating factors associated with their current or intended use of IBIs. Findings from the client groups clarified the barriers they experience in access existing face-to-face treatment services, which suggested that two primary reasons are behind clients’ decision to use IBIs, namely dissatisfaction with existing services and difficulty attending face-to-face treatment. As for clinician groups, findings showed that the primary motivator was a desire to reach clients experiencing barriers, which is influenced by a consideration of the advantages and disadvantages associated with providing treatment through IBIs.

### Part 1.1 clients

Clients revealed that the more they feel dissatisfaction with existing services, the more they are likely to consider IBIs as an alternative form of treatment. They cited lack of availability, lack of support during high-risk situations, lack of lived experience among service providers, and lack of access to professional support as factors that contribute to their dissatisfaction. Client responses also suggest that while most clients prefer professional guidance over peer support groups like Gamblers Anonymous (GA), professional guidance is significantly less accessible.

Client responses were valuable in gaining an understanding of the different barriers that they experience when seeking treatment. They reported barriers and challenges resulting into difficulty attending face-to-face treatment. This includes distance, transportation, timing constraints, waiting lists, financial challenges, feelings of shame and guilt, and implications of concurrent disorders. Table 2 illustrates these factors with example quotes from clients.

**Table 2:** Client motivations for using IBIs

Themes	Subthemes	Example quotes
Dissatisfaction with existing services	Lack of availability	“Just availability. More treatment offered in my area, more services … I think with addiction and mental health and gambling, it’s all such a big thing that’s going on, it affects people’s lives so much, and I feel like they should just have more services.”
	Lack of support during high-risk situations, including nighttime, weekends, and holidays	“A lot of the times the easiest time to get to a program is on the weekend and there’s hardly any programs here on the weekend.”
	Lack of lived experience among treatment providers	“There’s a lack of lived experience, in my opinion. It’s a lot of textbook, but there isn’t actually a person that has lived experience.” “It’s very theoretical rather than practical.”
	Preference for professional guidance over peer support groups	“I did like going [to GA sessions], but then it’s not so structured … There’s no professional counselor running them … There’s no structure to it.”
Difficulty attending face-to-face treatment	Distance to services	“I know that with a lot of people in small towns, there is absolutely nothing. I know someone right now that’s going through hell and can’t access anything within a hundred miles, so automatically this puts him in harm’s way.”
	Transportation	“For me, to travel for like an hour is really difficult, so for them to have more services in my area would be better.”
	Timing constraints	“A lot of people finish work at 5 pm so to get to group by 5:30 pm is quite difficult. I think if group started at 6 pm, a lot more people would be able to attend. A later time would be good.”
	Waiting lists	“There’s a huge waiting list. We’re talking about 2 or 3 months waiting time. I could’ve lost my house in 2 to 3 months.”
	Costs and financial constraints	“Downtown parking, it’s too much.”
	Feelings of shame and guilt	“A huge barrier for me is shame sometimes. If I haven’t been perfect or let’s say I missed a session with my therapist or my group or I missed a week … I’m embarrassed to come back.”
	Implications of concurrent disorders	“I think mostly it’s the weather and just being retired and lazy. And suffering from depression, I really find it hard to do anything.”

### Part 1.2 clinicians

Focus groups with clinicians demonstrated that many of them have some experience working with clients remotely—most commonly via Skype, phone, or email. It is not clear from the clinician responses whether these were sanctioned by the institution. Findings suggest that the clinicians’ desire to reach clients who are experiencing barriers is the primary motivator behind the decision to work with clients remotely.

Among the clinicians, there was consensus that IBIs can be beneficial in mitigating the negative impacts of barriers to treatment. However, clinicians were also quick to clarify that they do not perceive IBIs as a standalone service that would replace face-to-face treatment. Instead, IBIs were perceived as an adjunct service that can help mitigate the harms experienced by underserved populations, and as an opportunity to provide a more client-centred approach to treatment where clients are met in the context in which they live.

Clinicians were instrumental in understanding the advantages and disadvantages associated with engaging with IBIs by healthcare professionals. Advantages associated with IBIs were that in can reach clients experiencing barriers, promote client-centred care, free up time for clinicians, and increase uptake. Disadvantages associated with IBIs were that it can decrease trust due to anonymity, come with limitations of technology, and reduce quality of therapeutic work. Clinicians also discussed the possibility of IBIs posing unique barriers to clients who can’t afford an Internet connection or a computer. This list is reflected in Table 3 with example statements from clinicians.

**Table 3:** Advantages and disadvantages of IBIs according to clinicians

Themes	Subthemes	Example quotes
Advantages	Reaches clients experiencing barriers	“Can reach rural clients who don’t have transportation or access. ‘Cause if somebody lives a couple hours away from any service provider, that’s a significant barrier to coming in and accessing treatment.”
		“Sometimes my clients will say, ‘I can’t afford to come and see you. I don’t have gas money.’ And so it’s cheaper to [go online].”
		“[Can reach] people with health issues. ‘Cause I have a gentleman who has a lot of hip issues right now, and so I just do contact over the phone instead of him coming in cos that was a huge barrier for him.”
		“[Can reach people with] mental health issues too. Phobias.”
		“They might be more comfortable because of the stigma piece.”
		“I have several gambling clients that are in rural areas so they’re about an hour, an hour and a half away … and so I sit at my desk and remote in with them.”
	Promotes client-centred care	“It gives the clients the power, the opportunity to choose what they want, what they feel they need at that time.”
		“Could be anonymous if people want to.”
	Frees up time for clinicians	“It’s time for me. It’s like any other client that I know is going to be there just clicks on, boom, the client’s there, I see them, see the next client that’s in the waiting room. I don’t have to drive too. I don’t have travel time. I don’t have anything, really, except to sign in and it pops up.”
	Increases uptake	“I think it could generate more numbers for our programs if people could connect and do like an assessment or screening and then come in to see us.”
Disadvantages	Decreases trust	“Am I trusting without knowing who’s behind that name online?”
		“Confidentiality could be breached.”
	Comes with technological limitations	“You only see a proportion [on the screen].”
		“Sometimes technology fails us.”
	Reduces quality of therapeutic work	“Distractions from our end ‘cause I even find if I’m on the phone with a client, I might be sometimes multitasking, like, I’m not even focusing.”
	Comes with its own barriers	“Clients can’t always afford the Internet, or own a computer.”

### Part 2 factors that influence the acceptability of IBIs for problem gambling for both clients and clinicians

In this section, we used the theoretical framework of acceptability developed by Sekhon and colleagues [12], which is comprised of seven component constructs. The framework recognized the distinction between perceived acceptability and experienced acceptability, noting that acceptability can be assessed prospectively or retrospectively [12]. In this study, we asked clients and clinicians questions focused on their perceptions of acceptability of IBIs prior to any exposure to the intervention.

It should be noted that when the focus groups were held, we were in the design phase of the intervention and only two features were distinctly known to all focus group participants, namely that the intervention would be conducted over the Internet, and that it would be therapist-guided. Table 4 has a list of the constructs arranged alphabetically with corresponding definitions and example quotes from the focus group participants in each end user group.

**Table 4:** Factors that influence the acceptability of IBIs

Component construct	Description of the domain (D) and example quotes from client groups (C1) and clinician groups (C2)
Affective attitude	D: How an individual feels about the intervention C1.1: “I’d be very comfortable with it.” C1.2: “I’d be interested in a trial.” C2: “I’d be fearful of suicide ideation, how do you deal with that when that takes place? You don’t know who they are, where they are, and how to send help.”
Burden	D: The perceived amount of effort that is required to participate in the intervention C1: “If I thought it would be simple for me to do or somebody could do it for me, then yeah I could access it that way.” C2: “Definitely a lot of supervision or training on suicidal and homicidal thoughts. I feel like that’s really huge.”
Ethicality	D: The extent to which the intervention has good fit with an individual’s value system C1.1: “Accessibility, it’s everything. And something like this [could] maybe make a difference, right.” C1.2: “I want it to be confidential and private.” C2.1: “When I think about this, I don’t think about the physical barriers of distance or employment. I think about people who just wouldn’t be comfortable coming in to a treatment agency … And maybe who aren’t quite ready to actually walk in and take that ownership and do that face-to-face. We can give them something less threatening.” C2.2: “The success of our work is based on the relationship, and so if you take out components of that, then you’re increasing risk in the probabilities of success.”
Intervention coherence	D: The extent to which the participant understands the intervention and how it works C1: “I’d love to see that group, either a separate group online, but a closed group, not people stop by whenever they want like a Gamblers’ Anonymous... Maybe 10 if it’s online, and it’s the same people every day every certain time, but it’s online.” C2: “There are certain clients that maybe this can benefit or maybe it won’t benefit. So there can be some limitations in terms of what types of issues will be addressed.”
Opportunity costs	D: The extent to which benefits, profits, or values must be given up to engage in the intervention C1: “I wouldn’t want to share so much information. I would say just a limited amount of information. Just enough to get the help I need.” C2: “You lose that human connection.”
Perceived effectiveness	D: The extent to which the intervention is perceived as likely to achieve its purpose C1: “A lot of people in remote areas would be able to access CAMH. For the moment, those of us in Toronto with access to Toronto benefit, so people with mobility issues would be able to take part. So I think it would be excellent.” C2: “If the general population comes in but then there’s people who have barriers, we want to increase access to those people and this is how we do it, that seems to make sense to me.”
Self-efficacy	D: The participant’s confidence that they can perform the behaviour(s) required to participate in the intervention C1: “I’m sure I can learn. I don’t think it would be difficult.” C2: “I think for me, to start with, because I’ve never done it before, I want to kind of start from bottom-up. Like, narrow it and then widen it as I improve whatever that I need to do.”

Note: Component constructs were adopted from the theoretical framework of acceptability developed by Sekhon and colleagues [12]. The quotes above are a small sample of the transcript data and do not represent an exhaustive list of quotes

**Table 5:** Factors that increase the acceptability of IBIs for problem gambling

	Clients	Clinicians
Physical	Availability of services 24/7 Synchronous over asynchronous communication Therapist guidance Skills-focused programming Supports and services for loved ones	Closed sessions Video calling over text-only communication Good and reliable technology Basic and user-friendly technology Personalized messages Paperwork aid Tech support
Social	Integrated approach to treatment Privacy and data security	Policies and protocols Safety protocol ‘Netiquette’ Rigorous screening of clients Tiered approach to implementation Complete programming

### Part 3 factors that increase the acceptability of IBIs

Clients and clinicians identified a number of physical and social factors that can increase the acceptability of IBIs for problem gambling. We documented these factors as they were described by focus group participants.

### Part 3.1 clients

For clients, acceptability can be advanced by the integration of certain features, such as the availability of services on a 24-h basis. Clients also preferred programs with therapist guidance over purely self-help resources, and synchronous communication over asynchronous communication. Skills-focused programming that integrates the use of worksheets, homework and exercises, and guided meditation is also favoured by clients. Recognizing that gambling-related harms have an impact not just on the individual gambler but those around him or her as well, clients also identified the value in supports and services for loved ones, such as online forum discussions. As one client described:

“A lot of the time, by the time we get here, our families are like ‘yeah okay this is just another cycle.’ If this [online service] gets in, instead of making a whole trip there [or] here, maybe they only have to go online for a little bit to be able to get some of their vent out.”

Findings also show that an integrated approach to treatment is highly desired by clients. This was illustrated by the following exchange in the first client focus group:

“What I love about CAMH is that I have two addictions and mental health issues, and they’re able to treat all of them together. They [service providers] communicate.”

“Yeah, it’s actually a very good statement. I totally agree with that … The great thing is that you can get all the therapy within the same confines ‘cause lots of times there is crossover.”

### Part 3.2 clinicians

Clinicians described how the design and implementation of IBIs for problem gambling could be advanced by physical (e.g., closed sessions, video-based over text-based communication) and social (e.g., comprehensive safety protocol, rigorous screening process) factors.

Closed sessions, which was described as sessions having an element of start and end that can reinforce boundaries and structure, are preferred by clinicians. There was consensus among clinicians that platforms with an online face-to-face video component, also known as video calling, would work better than text-only communication models.

Noting their experiences of volatility with other Internet-based tools like Skype or Adobe Connect, clinicians emphasized the importance of good and reliable technology, including picture and audio quality. One participant spoke specifically about her experience with the problem gambling clients she sees, describing: “I find a lot of times when it gets complicated, people just get discouraged and then they stop. It just has to be basic and user-friendly.”

Another clinician spoke about her own problem gambling clients and their propensity for personalized messages: “I have a lot of clients that love those motivational emails a day.” To which, another member of the group added: “They really want to see what they’re doing, and they want to track changes, and they want to see their successes and failures as well.”

One clinician’s call for paperwork aid (“Can someone do our paperwork?”), including the automated scoring of screeners and assessment tools, generated laughs and endorsement from the group. Lastly, there was an agreement between clinician groups that tech support should be available whenever needed either by clients or clinicians: “Tech support available and ready to jump in while the session is going in”.

In terms of social factors, the development of policies and protocols was seen as a priority. This refers to a broad array of potential issues ranging from safety protocols to expectations from clients. As one clinician asked:

“Are we going to counsel somebody who accesses this service on their cellphone and they’re walking on the street and then they’re receiving counseling and they cross the street without looking where they’re going?”

Next, the clinicians also saw the value in developing group norms tailored for an online audience, which they called “netiquette instead of norms” – a play on Internet etiquette. This set of netiquette would also cover expectations of any client participating in an online group.

“I think expectations of the clients. So if it’s going to be more skill-based or structure-based, if they pop in for their video session and they haven’t done the worksheet that they’re supposed to have done … there’s only so much you can do around motivation … If they’re not going to put the work in, then it’s really not going to work.”

The development of a comprehensive exclusion criteria and rigorous screening for clients was another priority for clinicians. There was a consensus that if a client’s condition is severe, IBIs may not be the best fit.

“If their situation is severe, I’d say no. If it’s kind of mild and kind of assessing the safety stuff and all of that, I say yes. So, it depends on the client situation. I can’t say it would work for everyone.”

A number of clinicians raised the possibility of a tiered approach to implementation of IBIs wherein instead of treatment, dissemination would start with services deemed to be of lower risk, such as assessment, continuing care, or relapse prevention. Two clinicians from different groups shared their belief that IBIs would work best as a follow-up service. As one of them described: “In my experience, the only time it does work is when you have a really established relationship with someone and then they move, but you’ve already got the connection.”

Finally, clinician perspectives pointed towards the value of a complete programming for clients: “I think if somebody used the online service, it should be able to take them through their recovery. It should be a complete cycle. A complete program so to speak. That’s important.”

## Discussion

Although IBIs have been around for nearly two decades, evidence suggests a slow uptake of IBIs into clinical settings [16]. Increasing the acceptability of IBIs among end users may increase uptake [13]. Understanding acceptability alone is not enough to ensure successful uptake, however it can significantly impact overall effectiveness [12, 13, 16, 19, 24]. However, to date, research on IBIs has been largely focused on treatment outcomes in assessing effectiveness. While this is important, it offers little insight on how end users interact with interventions. This study is a step towards addressing this gap in the design and implementation of IBIs in health services. To do this, we used the theoretical framework of acceptability developed by Sekhon and colleagues [12], which recognized acceptability as a multi-construct concept. The assessment of perceived or prospective acceptability, another feature of the TFA, offers intervention developers the opportunity to design and implement an intervention that is informed by its target users.

One of the strengths of the study is in its juxtaposition of client and clinician perspectives. In general, clinician perspectives were more homogeneous reflective of healthcare professionals sharing the same practice and values. In comparison, client narratives were more heterogeneous descriptive of diverse experiences and individual preferences. Although both user groups reported similar experiences, each group also had unique concerns. For example, during the focus groups, clinicians repeatedly voiced the importance of clarifying the barriers experienced by clients seeking treatment and the utility of IBIs in addressing these barriers. The client focus groups were able to provide rich insight into the nature of the barriers they have experienced or are currently experiencing in accessing professional help. This was a strength of our study design as it allowed for the examination of each group’s perspectives adjacent to the other. Through this model, we were able to yield meaningful patterns across each end user group. Additionally, we were able to maximize the benefits associated with the use of focus groups in qualitative data collection, particularly its unique value in inciting group dynamics [22].

Clinicians and clients alike worried about the possibility of IBIs being a gateway to unintended use, such as Internet gambling. Although Internet gambling is a possible negative consequence of an IBI that should be monitored during intake and in each session, it should be noted that none of the participants in the client groups reported participating in Internet gambling. Involvement in Internet gambling should be taken into account when considering the client’s suitability for participating in IBIs, but it is not known if it should be an exclusion criterion. Whether online or Internet gambling can affect treatment outcomes for IBIs, and to what extent, warrants further investigation, perhaps in a sample of problem gamblers who only gamble online.

Concerns that IBIs would replace face-to-face treatment was repeatedly voiced by focus group participants from both end user groups. Although it was clarified in the beginning of each focus group that the purpose of the study was to investigate a “proof of concept,” participant responses often referred to a service that would replace current programming. This resulted in the facilitators repeatedly reminding participants, more so in the clinician groups, that the purpose of the study was to explore the feasibility of an online treatment service as an expansion of services to reach new clients who are heretofore not coming into treatment, rather than one that would replace existing face-to-face services. Nonetheless, some therapists may have been worried about losing their job to IBIs and exhibited signs of apprehension, which is consistent with the literature [25–27]. On the other hand, clients reported a strong preference for IBI run by therapists, rather than self-help resources. The literature remains inconclusive on the preference for IBIs by clients or clinicians, warranting further investigation.

On top of the intricacies involved in the design of IBIs in healthcare, the problem gambling population is unique and complex. Comorbidity with other psychiatric disorders is common [28, 29]. During the focus groups, some clients self-reported comorbid problem gambling and depression or anxiety problems. Two clients shared that their depression was making it difficult for them to attend treatment. It is therefore not surprising that an integrated approach to treatment was highly desired by the clients. These client perspectives are consistent with the results of a randomized controlled trial that examined the clinical efficacy of a computer-based psychological treatment for comorbid depression and substance use, which suggest that interventions that target both issues simultaneously can lead to better outcomes [3].

Researchers aiming to design effective interventions are faced with composite challenges. The findings from the focus groups are instrumental in understanding how best to approach the design of a problem gambling intervention. By asking end users probing questions focused on how they intend to interact with the intervention, we learned the specific factors that influence their decision to use IBIs. We also gained an understanding of the nature and extent of their past experience with Internet-based tools and resources for treatment, and how this influences their current treatment decisions. The clients and clinicians that participated in the focus groups offered various service user and clinical perspectives that may not be captured in the literature on IBIs. These perspectives are an important element of intervention studies that are complex in nature and comprise several interacting components for both treatment providers and treatment recipients. An intervention that meets both client and clinician needs and is considered acceptable, is more likely to guarantee the best treatment outcomes [12, 13, 16, 19, 26].

## Limitations

The study sample was not evenly distributed as the clinicians outnumbered the clients who participated in the focus groups. This may be attributed to challenges recruiting among problem gamblers that have been observed by other researchers [30]. The majority of clients that were eventually recruited were clients at the Problem Gambling Services at CAMH comprised of service users who may be more motivated or engaged with mental health services. Additionally, all of the clients who participated in the focus groups were adults living in urban areas in one province in Canada. As a result, the narratives from problem gamblers living in rural areas may not have been captured. Research drawing upon a representative sample of problem gamblers in Canada is needed. To reduce participant burden, the research team made conscious efforts to minimize the need for participants to provide any personal information, including demographic information. Methodological limitations must also be mentioned. Focus groups, by tapping into interpersonal communication and group dynamics, offer a unique opportunity to explore issues that are not well understood or have received little attention in the past. However, focus groups also pose unique challenges for participants who may not be assured confidentiality in a group setting, may feel reluctance to discuss sensitive topics in a group, or may be affected by common issues related to focus groups like groupthink or response bias [21]. Ultimately, the objectives of the focus groups were met as can be determined from the data collected, which provide insight into the acceptability of IBIs for clients and clinicians.

## Conclusions

This study provides insight into the factors that influence the acceptability of IBIs among end users, including access, usability, high quality technology, privacy and security, and professional guidance. Acceptability is an important factor in the overall effectiveness of IBIs. Gaining an understanding of how end users perceive IBIs and why they choose to use IBIs can be instrumental in the successful and meaningful design, implementation, and evaluation of IBIs. Findings from the focus groups have implications for others developing IBIs for problem gambling and other similar issues.

## Data Availability

Following the approved ethics protocol, the raw qualitative data collected in this study may not be shared to protect participants’ privacy and integrity.

## Abbreviations

CAMH: Centre for Addiction and Mental Health
DHI(s): Digital health intervention(s)
IBI(s): Internet-based intervention(s)
TFA: Theoretical framework of acceptability
